# Measuring Female Gaming: Gamer Profile, Predictors, Prevalence, and Characteristics From Psychological and Gender Perspectives

**DOI:** 10.3389/fpsyg.2019.00898

**Published:** 2019-04-26

**Authors:** Olatz Lopez-Fernandez, A. Jess Williams, Daria J. Kuss

**Affiliations:** ^1^Department of Psychology, International Gaming Research Unit, Nottingham Trent University, Nottingham, United Kingdom; ^2^Turning Point, Eastern Health and Clinical School, Monash University, Melbourne, VIC, Australia; ^3^School of Psychology, Institute for Mental Health, University of Birmingham, Birmingham, United Kingdom

**Keywords:** internet addiction, internet gaming disorder, gaming disorder, female gender, female gamer, psychopathology, psychological assessment, psychometrics

## Abstract

Research investigating female gaming is relatively scarce, and past research has demonstrated that men are more likely to be problematic gamers. Few studies have focused on female gamers in community samples, and those that have been published have mainly collected qualitative data in Europe. There is case study evidence suggesting clinicians are increasingly treating problem female gamers. The aim of this study is threefold: (i) to establish an international female gamer profile, (ii) to determine predictors associated with perceived internet gaming disorder (IGD), and (iii) to identify those who are potentially at risk of developing gaming addiction and its characteristics by applying a quantitative approach. A cross-sectional online survey was applied through international gaming forums recruiting 625 female gamers, assessing sociodemographics, gaming devices used and play genres, and a set of questionnaires on gaming [e.g., problem online gaming (e.g., the nine-item short-form scale to assess IGD: IGDS9-SF), female stereotypes (e.g., sex role stereotyping scale), and psychological symptoms (e.g., Symptom CheckList-27-plus)]. Female gamers from all continents reported the use of all videogames, especially popular online games using computers and consoles. The proportion of gamers with potential IGD was one per cent. Regression analyses identified several risk factors for increased scores on the IGDS9-SF, namely having achievement and social motivations, embodied presence and identification with the avatar, hostility and social phobia together with negative body image, playing Multiplayer Online Battle Arena games, Massively Multiplayer Online Role-Playing Games, and First-Person-Shooter games. Findings contribute to filling the gap in knowledge on female gaming, to aid in the applicability of problematic gaming measurements in female gamers, especially those who are at risk of problematic gaming. The purpose of this study is to enhance the validity of the current measures to diagnose problem gaming appropriately in both genders.

## Introduction

Female gaming research is relatively scarce (Lopez-Fernandez, 2018), and it has demonstrated that men are more likely to be problematic gamers. One of the reasons for this discrepancy is traditionally videogames were designed by males for males (Kuss and Griffiths, 2012). Another reason is the persistence of the male gamer stereotype, which may negatively reflect on females, who are not yet considered “real” or “hardcore” gamers. This contention is supported by professional and highly visible figures in gaming culture who are usually males (Paaßen et al., 2017). Thus, instead of being recognized as skilful and resourceful gamers themselves, female gamers are often discriminated against (Vermeulen et al., 2017), leading to a female gamer identity tainted by perceived threat and stigmatization (Matthews et al., 2016). This is exacerbated by female avatars being overly sexualized and women being objectified, which is particularly the case for fighting games (Lynch et al., 2016).

These psychosocial factors place women outside of the international gaming culture, while recent studies addressing problematic gaming in both genders find female gamers may be at risk for problematic gaming behaviors (Laconi et al., 2017; Lopez-Fernandez, 2018). When investigating the impact of playing specific games on online gaming addiction, in the more popular games [e.g., Massively Multiplayer Role-Playing Games (MMORPGs), Multiplayer Online Battle Arena (MOBA), and Real-Time Strategy (RTS) games] both genders score high on addiction measures (Kuss et al., 2012; Eichenbaum et al., 2015; Fuster et al., 2016; Laconi et al., 2017). However, the research on gaming addiction within female populations indicates several variables contribute to this behavior: older age, lower self-esteem and life satisfaction, higher depression, and higher chances of getting into serious fights (Ko et al., 2005; Desai et al., 2010; Laconi et al., 2017). These findings suggest that contrary to boys, externalizing behaviors may be present in girls who play online games, further supporting the contention that gender appears to play an essential role in online gaming and possible addictive use. Nevertheless, it seems young male gamers are more susceptible to problematic gaming than female gamers (Wu et al., 2013), which may explain the focus on male gamers in contemporary research on problematic gaming.

The few existing papers about female gamers research factors associated with gender-related problems, and have linked this to experiences of aggressive and sexualized behaviors in virtual and real worlds. Eastin (2006) showed women experience higher involvement in the game and more aggressive thoughts when their game character has the same gender as themselves (i.e., both are female). Kaye et al. (2017) demonstrated avatar gender has an impact on how competent a player is considered to be (e.g., female gamers playing male avatars were found to be more highly skilled than those with female characters; an effect which does not appear with male gamers); similarly, women tend to have more aggressive thoughts when playing against a player of the opposite gender (e.g., a male player). Behm-Morawitz and Mastro (2009) observed playing a sexualized game heroine (e.g., where the female body is exposed with larger breasts and a smaller waist) negatively influences people’s beliefs about female gamers in the offline world.

The objectification of women and female avatars in games has contributed to the overly sexualized representation of females in games during the last three decades, despite the perception of women being secondary to men in terms of their skills in gaming (Lynch et al., 2016). Barlett and Harris (2008) suggested the representation of one’s avatar in the game may have a negative impact on body image, to the extent of developing body dissatisfaction (Martins et al., 2009) and leading to decreased self-esteem, feelings of inferiority, and depression. This is similar to negative downward comparisons of the self with others on visually focused social networking sites (e.g., Facebook, Instagram, and Snapchat), which has been linked to the experience of poorer mental health (e.g., depression), body image concerns, and addictive use (Donnelly and Kuss, 2016; Marengo et al., 2018). Therefore, it appears plausible to suggest female gamers may be at risk for developing body dissatisfaction as a consequence of playing female avatars which tend to be oversexualized in the average videogame.

Few studies have been published concerning female gamers in Europe (e.g., Eklund, 2011; Lewis and Griffiths, 2011; McLean and Griffiths, 2013; Shaw, 2011). Findings from these qualitative studies have shown women usually play casual games, typically for shorter periods compared with men, although half of these studies (Eklund, 2011; Lewis and Griffiths, 2011) also state female gamers play MMORPGs. These studies, however, have not explored why women play videogames, stating they only recognize they are “wasting time for gaming” (Lewis and Griffiths, 2011). To the best of the present authors’ knowledge, the connection between excessive time spent playing videogames, the game genres played, and the potential of developing addiction problems in female gamers remains unknown.

Furthermore, gaming motivations appear to differ cross-culturally across genders. European research shows apart from escapism, females also look for competition [e.g., for the pleasure of defeating others (Laconi et al., 2017) or challenging themselves (Lewis and Griffiths, 2011)]; while males look for coping [i.e., to deal with stress (Laconi et al., 2017)], and success [e.g., through competition (Demetrovics et al., 2011)]. However, Asian research (Ko et al., 2005) indicates females play for achievement and social reasons, while males play to pass the time. In America, it seems female gamers look for achievement and power, to engage socially and maintain relationships (Taylor, 2006). This latter motivation has been associated with social gaming in romantic partner relationships and has been linked to greater happiness and healthier gaming in females relative to their male partners (Williams et al., 2009), suggesting gaming motivations impact on gaming experience and perceptions, which may be linked to problematic gaming. Overall, females have different gaming motivations than males.

According to recent statistics, over 45% of Western gamers are now estimated to be female (Entertainment Software Association [ESA], 2018; Interactive Software Federation of Europe [ISFE], 2018). Despite this, scientific knowledge of female gaming is surprisingly scarce. There is case study evidence suggesting clinicians are increasingly treating female gamers; psychotherapists and psychiatrists treating Internet Gaming Disorder (IGD; American Psychiatric Association [APA], 2013) and Gaming Disorder (World Health Organization [WHO], 2018) indicate it may go unnoticed in females (Kuss and Griffiths, 2015), who also show psychopathological differences connected to their addiction compared with men (González-Bueso et al., 2016). Indeed, based on the IGD proposal, it has been stated the official criteria should be valid across genders (Petry et al., 2014). However, only a few studies on this behavioral problem have focused on female populations, given the majority of MMORPGs gamers who have been associated with an increased risk of developing addictions are males (Kuss, 2013b; Lopez-Fernandez et al., 2014). In addition to this, male gamers are significantly more likely to appear in the clinical context seeking support for their gaming-related problems (Kuss and Pontes, 2019; Lopez-Fernandez and Kuss, 2019).

Taken together, our current knowledge of female gaming is limited, particularly with regards to problematic gaming and associated symptoms. Given that female gaming experiences, motivations and perceptions appear to be different from those of males, problems associated with excessive gaming may be experienced differently relative to male gamers, who scientific studies have focused on. The present research aims to fill the gap in knowledge on female gaming by (i) establishing international female gamer profiles, (ii) determining potential predictors associated with perceived IGD, and (iii) identifying female gamers who may be at risk for developing online gaming addiction and associated characteristics using a quantitative research approach.

## Materials and Methods

### Participants and Procedure

An international sample of individuals who play videogames was surveyed (see Table 1). Regarding the personal characteristics of the sample, the majority were employed or full-time students, nearly all had a high level of education (secondary and higher education), and their cultural background was from Western cultures (i.e., America and Europe).

**Table 1:** Sociodemographics of female gamers [*N* = 625; qualitative variables are shown with valid percentages and quantitative variables include means (*M*), and standard deviations (*SD*)].

Female gamers *N* = 625	
Variables values	% /*M (SD)*
**Profession**	
Employed full-time	37.9
Student	33.9
Unemployed	10.2
Other (Employed part-time, Student and employed, Disable, Carer, etc.)	27
**Educational level**	
Higher education (Bachelor, Master, and PhD)	55.6
Secondary education (High school, sixth form, vocational training)	43.2
Primary education (School, apprenticeship, or unspecified)	1.3
**Continent**	
America	49
Europe	42.2
Africa	3.8
Asia	2.9
Oceania	1.4
**Age**	*26.87 (6.9)*

*Profession: Participants were asked to select more than one, and were therefore included more than once; e.g., student and employed; Continents (Countries): America (United States, Canada, Brazil, Peru); Europe (United Kingdom, Germany, Netherlands, Finland, Poland, France, Italy, Sweden, Romania, Austria, Czechia, Portugal, Denmark, Greece, Hungary, Norway, Spain, Belgium, Latvia, Russia, Switzerland, Croatia, Lithuania, Luxembourg, Malta, Serbia, Slovakia, Slovenia, Turkey); Africa (Nigeria); Asia (Singapore, India, South Korea, Hong Kong, Indonesia, Iraq, Israel, Japan, Thailand, United Arab Emirates); Oceania (Australia, New Zealand). Italic values refer to means.*

Online posts on social media, specific gaming forums, and related Reddit sub-forums aided recruitment. Moderators were approached for permission to post within closed groups or posts were ensured to meet the rules of the forum on open online communities, as put forward by the British Psychological Society [BPS] (2017) Ethics Guidelines for Internet-Mediated Research. Posts specifically called for female gamers to take part, but men could complete the survey if they so wished. All recruitment methods provided an anonymous link to the online survey, which ran from May–July 2018. Participants were informed that if they left contact details, they would be entered into a lottery draw and invited to interview for the second part of this study.

A total of 1,069 responses were received. However, responses were removed for a number of reasons: the response was part of the pilot study, had an odd behavior when responding (e.g., responding with the same answers to all questions), or the participant disengaged prior to finishing the demographic questions (i.e., informed consent statements) (*n* = 366). This resulted in a final sample for analysis of 703 respondents. However, only those who identified as female were analyzed (*N* = 625), with a mean (*M*) age of 26.87 [standard deviation (*SD*) = 6.9].

Permission to conduct this study was obtained from the School of Social Sciences’ ethical review board at Nottingham Trent University (United Kingdom). Informed consent within the online survey, in its first webpage (i.e., sociodemographic questions), through confirming seven questions (e.g., “I understand the study and agree to take part in it,” “I understand my participation is voluntary, and that I am free to withdraw (…),” “I confirm that I am a woman,” “I confirm that I am 18(…),” “I confirm that I play video games,” “I confirm that I know full participant anonymity will be assured (…),” and “I know that anonymized results may also be disseminated in academic work”).

### Measures

#### Demographics

This information was split by basic information such as age (in years), gender (female, male, and other), which country the response came from, among other variables (see Table 1), and descriptive gaming information (see Tables 2, 3). These gaming questions focused on typical habits (i.e., platform use, types of games played, and when participants played). This section included questions regarding time spent playing in a day (in hours), and time spent playing different game genres [i.e., asking female gamers about their perceived percentage of time playing first-person shooter (FPS), MMORPGs, MOBA or role-playing games (RPG), among other game genres]. Concerning the perceived violence in games and avatars by gender, questions were adapted from a previous study investigating violence in videogames (Anderson and Dill, 2000). Additionally, a question was added, which asked participants to rate the avatars’ bodies from realistic (1) to very exaggerated (5) (Matthews et al., 2016) with the added caveat for when a character was clearly not a human (6). These questions were included as they were deemed important themes by a related systematic review (Lopez-Fernandez et al., unpublished).

**Table 2:** Patterns of playing video games [*N* = 625; qualitative variables are shown including valid percentages with a proportion higher than 40%, and quantitative variables include means (*M*), and standard deviations (*SD*) with a mean higher than one].

Female gamers (*N* = 625)		
Variables (Values)	%	*M (SD)*
**When do you usually play?**		
Weekend and weekdays	59.7	
**Which gaming platforms do you usually play on?**		
PC online games	53.6	
PC offline games	51.5	
Console offline games	44.3	
**How many platforms do you usually use for gaming?**		*2.10 (1.02)*
**How many hours do you usually play per day?**		*3.42 (2.60)*
**Percentage of time spent on video games**		
FPS (e.g., Call of Duty)		*13.75 (22.87)*
MMORPG (e.g., World of Warcraft)		*18.60 (27.24)*
MOBA (e.g., League of Legends)		*4.27 (12.97)*
Role play (e.g., Final Fantasy)		*18.88 (22.14)*
Simulations (e.g., The Sims)		*10.46 (18.15)*
Real-time strategy (e.g., Warhammer)		*1.92 (6.84)*
Casual games or apps (e.g., Candy Crush)		*6.9 (14.27)*
Action adventure (e.g., Uncharted)		*10.35 (16.46)*
Adventure point and clicks (e.g., Monkey Island)		*1.81 (5.46)*
Platformer (e.g., Super Mario)		*3.56 (9.37)*
Puzzle (e.g., Tetris)		*2.97 (7.92)*
**How often do you play video games with someone else in a face-to-face context?**		
Never and rarely	52.5	
**How often do you play video games with someone else online?**		
Sometimes and very often	46.8	
**Who first introduced you to play video games?**		
Family member (e.g., Father, Older brother, Friend, Mother, Cousin, Uncle, Aunt, Grandfather, etc.)	55.3	

*PC, personal computer; FPS, First-Person Shooter; MMORPG, Massively Multiplayer Online Role-Playing Game; MOBA, Massively Online Battle Arena. Italic values refer to means.*

**Table 3:** Contents of video games according to participants’ accounts (*N* = 625; valid percentages of the valued with a proportion higher than 20%).

Female gamers *N* = 625	
Endorsement	%
**How violent is the content of your favorite game?**	
Somewhat above average	26
Average	37.3
**How often is violence against women shown in the game?**	
Average	35.1
Far below average	27.9
**How often is violence against men shown in the game?**	
Average	38.8
**Rate the bodies of the main characters featured within the game?**	
Very exaggerated	30.4
Quite exaggerated	23.4

#### Internet Gaming Disorder Scale – Short Form (IGDS9-SF; Pontes and Griffiths, 2015)

This scale measured the participants’ gaming activity during the last 12 months and its impact on aspects of the person’s life. High scores (36/45 points) are deemed indicative of a higher degree of IGD, with at least five of the nine criteria being endorsed as very often. Response options were “Never” (1) to “Very often” (5), with a minimum score of 9 and a maximum of 45. Cronbach’s alpha for the IGDS9-SF was 0.86.

#### Online Game Motivation Scale (OGMS; Yee et al., 2012)

Participants were asked to rate the importance of game play elements through three domains (with four items each; overall 12 questions): immersion motivation (e.g., “feeling immersed in the world”), social motivation (e.g., “being part of a guild”), and achievement motivation (e.g., “becoming powerful”) in gaming behavior. Response options ranged from “Not important at all” (1) to “Extremely important” (5). Cronbach’s alpha for the OGMS in this sample was 0.77. Additionally, the alphas for the subscales ranged from 0.64 to 0.90.

#### Embodied Presence (EP; Van Looy et al., 2012)

It is a subscale of six questions from Van Looy et al. (2012) three-factorial structure of identification in Massively Multiplayer Online Games (MMOGs). These questions considered how connected the respondents feel to their own videogame avatar (i.e., avatar identification; e.g., “I feel like I am inside my character when playing”). Response options ranged from “Strongly disagree” (1) to “Strongly agree” (5). In this sample, Cronbach’s alpha was 0.93.

#### Antecedents of Identification (AoI; Cohen, 2001)

Ten questions were adopted to relate to avatars and experiences when playing MMORPGs or MOBA games. These included statements such as “During play, I feel as though I could really get inside my character’s head” and “While playing MMORPGs/MOBAs, I forget myself and I am fully absorbed.” Response options ranged from “Strongly disagree” (1) to “Strongly agree” (5). In this sample, Cronbach’s alpha was 0.91.

#### Coping Strategies (CS; Fox and Tang, 2017)

The subscale of gender masking was adapted from the identified strategies women use to cope with online harassment within games (Fox and Tang, 2017). The scale’s six items were indicative of strategies that would make women less likely to experience gender-specific harassment (e.g., “Use a male avatar”). Response options ranged from “Extremely unlikely” (1) to “Extremely likely” (5). Cronbach’s alpha in the present sample was 0.87.

#### Symptom Checklist-27-Plus (SCL-27-Plus; Hardt, 2008)

The SCL-27-plus was selected over the original Symptom Checklist-90 (Derogatis et al., 1976). To be relevant for gaming disorder, the hostility (e.g., “feeling easily annoyed or irritated”), agoraphobia (e.g., “Fear to leave the house alone”), social phobia (e.g., “Fear to say something embarrassing”), and depression after a year (e.g., “Melancholy”) subscales were included. Response options ranged from “Never” (0) to “Very often” (4). The overall Cronbach’s alpha was 0.93. Additionally, the alphas for the subscales ranged from 0.80 to 0.91.

#### Body Shape Questionnaire-8c (BSQ-8c: Evans and Dolan, 1993)

This scale was selected due to its reduced length (i.e., eight items; Evans and Dolan, 1993; e.g., “Have you imagined cutting off fleshy areas of your body?”) in comparison to the 34-item BSQ (Cooper et al., 1987). Body shape information was important given the negative influence that videogame characters’ appearance has been indicated to have on gamers (Barlett and Harris, 2008). Response options ranged from “Never” (1) to “Always” (6). Cronbach’s alpha was 0.93.

#### Bem Sex Role Inventory – Short Form [BSRI – Short Form (BSRI-SF): Bem, 1981]

The BSRI-SF was used to determine to what extent personality attributes attached to stereotypical masculinity (BSRI-SF-m; e.g., “assertive,” “forceful,” and “aggressive”) and femininity (BSRI-SF-f; e.g., “gentle,” “sympathetic,” and “loves children”) were identified with. Response options ranged across a 7-point scale from “Never or almost never true” to “Almost always true.” The overall Cronbach’s alpha reached 0.86 (masculine 0.87, feminine 0.90).

### Statistical Analyses

IBM SPSS version 25 for Windows (IBM Corp. Released, 2017) was used and statistical analyses used a significance level of *p* < 0.05. Descriptive statistics were carried out for sociodemographics, usage patterns of playing videogames, type of games, and descriptive item analysis. Cronbach’s alpha coefficients were used to estimate reliabilities. Pearson’s correlation coefficient was calculated with effect sizes based on Cohen et al. (2003), where *r*^2^ ≥ 0.01 denotes low, *r*^2^ ≥ 0.03 medium and *r*^2^ ≥ 0.05 high effects. One-way analyses of variance were performed to examine the effect of gaming addiction in female gamers. Multiple linear regression analyses were undertaken with gaming addiction as outcome variable: (i) online gaming addiction was predicted by the other psychological and gender characteristics, (ii) online gaming addiction and the different videogame genres studied. To estimate the prevalence of potential problem users with IGD, the proposed cut-off scores were considered (Petry et al., 2014; Pontes et al., 2017). Lastly, the data from the “addicted” groups of gamers were considered in more detail to identify a specific profile.

## Results

### Female Videogame Playing

Female gamers usually played “popular online games” (see Table 2), presented according to time spent gaming, from higher to lower: RPGs, MMORPGs, FPSs, simulations, action adventure, MOBAs, and other games (e.g., sports or education games). Participants reported using different platforms to play, usually computers and consoles. However, almost all “popular games” (e.g., FPSs, MMORPGs, and MOBAs) were preferably played using PCs online rather than offline, and also via online consoles; while “small games” were usually played through apps or mobile games (i.e., casual games and educational games). For instance, MMORPGs and MOBAs were preferably played on a PC online [χ^2^_(1)_ = 129.5, *p* < 0.001; *V* = 0.48, *p* < 0.001; χ^2^_(1)_ = 31.3, *p* < 0.001; *V* = 0.24, *p* < 0.001], while adventure point and click games or RPGs were usually preferentially played on a PC offline [χ^2^_(1)_ = 45.62, *p* < 0.001; *V* = 0.28, *p* < 0.001; χ^2^_(1)_ = 24.49, *p* < 0.001; *V* = 0.21, *p* < 0.001]. Regarding consoles online, only sports games were slightly preferred [χ^2^_(1)_ = 8.6, *p* < 0.01; *V* = 0.12, *p* < 0.01], and offline action adventure [χ^2^_(1)_ = 51.01, *p* < 0.001; *V* = 0.30, *p* < 0.001] or platformer games [χ^2^_(1)_ = 41.79, *p* < 0.001; *V* = 0.27, *p* < 0.001] were preferably played. In the case of apps (i.e., mobile games), casual games [χ^2^_(1)_ = 184.31, *p* < 0.001; *V* = 0.57, *p* < 0.001] and education games [χ^2^_(1)_ = 15.68, *p* < 0.001; *V* = 0.17, *p* < 0.001] were the only ones reported. Concerning frequency of time spent on videogames, only a fifth of female gamers tended to play with someone else in a face-to-face context, whereas almost half of these players usually play online with other gamers. Participants played several times a week, and a quarter of them played daily (24.6%). Interestingly, only a quarter discovered gaming by themselves, as the majority was introduced to it by others, usually a male relative (e.g., father or older brother).

### Female Gaming and Violent Content

Only a third considered their favorite videogame to have violent content, which was usually the case for MMORPGs and MOBAs, and only 12% reported violence against women in these games, while a quarter considered there was violence against males. Half of the female gamers considered the bodies of the main characters within the games they played as being exaggerated (see Table 3).

### IGD in Female Gamers: Characteristics, Correlations, and Predictors

With respect to sociodemographic measures, the level of education achieved was significantly different regarding IGD [*F*_(4,546)_ = 2.541, *p* < 0.05]. Ordered in terms of their strongest predictive power [*M*(*SD*)] higher education was the weakest predictor, being the most relevant: secondary education [19.26(6.74)], and primary education [18.33(4.93)]. Similarly, the type of occupation [*F*_(4,509)_ = 3.958, *p* < 0.01] showed those with more free time tend to present higher IGD scores: unemployed [18.69(6.31)], students [18.20(6.27)], part-time employed [17.83(6.91)]. Lastly, residence on specific continents presented statistical differences in relation to IGD [*F*_(4,546)_ = 4.358, *p* < 0.01], especially in Asia [21.43(8.39)], and Africa [19.19(7.07)].

Five-hundred-and-three participants completed the IGD and had a low mean score (i.e., an average of 17 out of 45), which was positively associated with almost all measures (i.e., gaming motivations, embodied presence, avatar identification, psychopathology, and body shape), but the effect ranged between medium to small (see Table 4). For instance, IGD and hostility had higher correlations, but with medium effect sizes (*r*^2^_hostility_ = 0.17), while IGD and participants’ beliefs regarding stereotypical gender roles had a low correlation with a small effect size (*r*^2^_hostility_ = 0.02). Moreover, no association was found between potential IGD and the personality attributes attached to stereotypical masculinity and femininity characteristics.

**Table 4:** Descriptive (mean and standard deviation), reliability (Cronbach’s alpha), and correlation matrix (Pearson’s r) of the scales completed by female gamers.

Scale	M *(SD)*	α	IGDS9-SF9	OGMS i	OGMS s	OGMS a	EP	AoI	CS	SP-27-p h	SP-27-p a	SP-27-p sp	SP-27-p d	BSQ-8c	BSRI m	BSRI f
IGDS9-SF9	17.31 (6.05)	0.86	1													
(*n* = 553)																
OGMS i	14.97 (3.66)	0.80	0.11^∗^	1												
(*n* = 548)																
OGMS s	10.89 (3.13)	0.90	0.25^∗∗^	0.03	1											
(*n* = 548)																
OGMS a	8.59 (4.35)	0.64	0.32^∗∗^	0.00	0.36^∗∗^	1										
(*n* = 548)																
EP	15.64 (6.72)	0.93	0.24^∗∗^	0.35^∗∗^	0.03	0.17^∗∗^	1									
(*n* = 536)																
AoI	31.12 (8.54)	0.91	0.25^∗^	0.35^∗∗^	0.21^∗∗^	0.18^∗∗^	0.45^∗^	1								
(*n* = 536)																
CS	13.48 (6.23)	0.87	0.08	0.06	−0.11^∗∗^	0.00	0.02	0.04	1							
(*n* = 536)																
SP-27-p h	3.48 (3.40)	0.80	0.41^∗∗^	0.04	−0.03	0.20^∗∗^	0.15^∗∗^	0.08	0.06	1						
(*n* = 523)																
SP-27-p a	3.27 (3.91)	0.88	0.31^∗∗^	0.13^∗∗^	0.05	0.09	0.16^∗∗^	0.1^∗^	0.09	0.28^∗∗^	1					
(*n* = 523)																
SP-27-p sp	7.98 (5.73)	0.91	0.40^∗∗^	0.15^∗∗^	0.04	0.09	0.19^∗∗^	0.15^∗∗^	0.13^∗∗^	0.38^∗∗^	0.68^∗∗^	1				
(*n* = 523)																
SP-27-p d	9.03 (1.43)	0.80	0.23^∗∗^	0.14^∗^	−0.00	0.04	0.19^∗∗^	0.15^∗∗^	0.05	0.2^∗∗^	0.27^∗∗^	0.4^∗∗^	1			
(*n* = 523)																
BSQ-8c	21.43 (10.62)	0.93	0.32^∗∗^	0.14^∗∗^	0.06	0.12^∗∗^	0.1^∗^	0.1^∗^	0.14^∗∗^	0.26^∗∗^	0.32^∗∗^	0.44^∗∗^	0.27^∗∗^	1		
(*n* = 519)																
BSRI m	51.14 (10.58)	0.87	−0.3	0.11^∗^	0.10^∗^	0.03	0.1^∗^	0.04	0.01	−0.11^∗^	−0.25^∗∗^	−0.27^∗∗^	−0.08	−0.04	1	
(*n* = 519)																
BSRI f	43.33 (10.46)	0.90	−0.7	0.13^∗∗^	0.14^∗∗^	0.19^∗∗^	0.1^∗^	0.18^∗∗^	0.01	0.05	0.01	−0.04	0.04	0.05	0.13^∗∗^	1
(*n* = 516)																

*^∗^p < 0.05, ^∗∗^p < 0.001; IGDS9-SF9, Internet Gaming Disorder Scale – Short Form; OGMS, Online Game Motivation Scale [i, immersion; s, social; a, achievement EP, Embodied Presence; AoI, Antecedents of Identification; CS, Coping strategies; SP-27-p, Symptom Checklist-27-Plus (h, hostility, a, agoraphobia, sp, social phobia; d, depression last year); BSQ-8c, Body Shape Questionnaire-8c; BSRI, Bem Sex Role Inventory – short form (m, masculine, f, feminine)].*

Similarly, IGD had significant positive correlations with FPSs (*r* = 0.12, *p* < 0.01), MMORPGs (*r* = 0.11, *p* < 0.05), MOBAs (*r* = 0.15, *p* < 0.01); and significant negative correlations with RPGs (*r* = −0.13, *p* < 0.01), simulation games (*r* = −0.10, *p* < 0.05), and adventure point and clicks games (*r* = −0.09, *p* < 0.05). The effect sizes were all small (e.g., MMORPG *r*^2^ = 0.01).

All regression analyses were run in 514 participants who completed all questionnaires and variables were included as predictors. The variance inflation factor (VIF) and tolerance index supported the absence of multicollinearity (i.e., gaming addiction predicted by psychological characteristics: VIF_max_ = 2.34, tolerance_min_ = 0.43; gaming addiction explained by game genres: VIF_max_ = 4.92, tolerance_min_ = 0.20). The Durbin–Watson coefficients indicated a lack of autocorrelation between adjacent residuals in both analyses (i.e., gaming addiction with characteristics: 0 < 1.87 < 4; gaming addiction with games: 0 < 1.31 < 4). The first model explained 37.1% of variance in IGDS9-SF scores [*R*^2^ = 0.371; *F*_(10,508)_ = 30.639, *p* < 0.001], and showed that IGD is best predicted by (1) achievement motivation, (2) social motivation, (3) embodied presence, (4) avatar identification, (5) hostility, (6) social phobia, and (7) body shape perception, in that order. The second model only explained 6.5% of variance in IGDS9-SF [*R*^2^ = 0.065; *F*_(6,540)_ = 6.282, *p* < 0.001], and emphasized that online gaming disorder is best predicted by playing (1) MOBAs, (2) FPSs, and (3) MMORPGs (see Table 5).

**Table 5:** Constructs and video game genres regressed on potential gaming disorder (IGDS9-SF).

		Female gamers who completed the measures (*N* = 514)				
Outcome variables	Predictor	*B*	*SE B*	*t*	β	*p*
Gaming addiction	OGMS achievement	0.32	0.08	4.214	0.17	<0.001
(IGDS9-SF)	OGMS social	0.21	0.05	3.968	0.15	<0.001
	Embodied presence	0.08	0.04	2.073	0.09	<0.05
	Antecedents of identification	0.07	0.03	2.339	0.10	<0.05
	SP-27-p hostility	0.45	0.07	6.58	0.26	<0.001
	SP-27-p social phobia	0.19	0.06	3.336	0.18	<0.01
	BSQ-8c	0.07	0.16	1.254	0.05	<0.01
	FPS	0.03	0.01	2.467	0.12	<0.05
	MMORPG	0.02	0.01	2.178	0.11	<0.04
	MOBA	0.07	0.02	3.328	0.15	<0.01

*IGDS9-SF, Internet Gaming Disorder Scale – Short Form; FPS, First-Person Shooter; MMORPG, massively multiplayer online role playing; MOBA, massively online battle arena; OGMS, Online Game Motivation Scale; SP-27-p, Symptom Checklist-27-Plus; BSQ-8c, Body Shape Questionnaire-8c.*

According to Pontes and Griffiths (2015), to differentiate disordered gamers from non-disordered gamers DSM-5 criteria should be used (Petry et al., 2014; see Table 6).

**Table 6:** IGD criteria endorsed by female gamers (*N* = 553; valid percentages).

Female gamers	
*N* = 553	
Variables	% endorsement
**IGD – Items**	
(1) Do you feel preoccupied with your gaming behavior? (Some examples: Do you think about previous gaming activity or anticipate the next gaming session? Do you think gaming has become the dominant activity in your daily life?)	8.7
(2) Do you feel more irritability, anxiety or even sadness when you try to either reduce or stop your gaming activity?	1.8
(3) Do you feel the need to spend increasing amount of time engaged gaming in order to achieve satisfaction or pleasure?	3.1
(4) Do you systematically fail when trying to control or cease your gaming activity?	1.4
(5) Have you lost interests in previous hobbies and other entertainment activities as a result of your engagement with the game?	1.6
(6) Have you continued your gaming activity despite knowing it was causing problems between you and other people?	2
(7) Have you deceived any of your family members, therapists or others because the amount of your gaming activity?	1.8
(8) Do you play in order to temporarily escape or relieve a negative mood (e.g., helplessness, guilt, anxiety)?	10.1
(9) Have you jeopardized or lost an important relationship, job or an educational or career opportunity because of your gaming activity?	0.7
**IGD – Prevalence**	1.1

### Profiling Potentially Addicted Female Gamers

Based on the conservative cut-off as delineated in the section above, only one percent of the sample was considered to have potential IGD (six participants).

This small sub-sample was between 18 and 32 years old. Their professional status was similarly diverse (two employed, two students, one unemployed, and another one work-disabled). The educational status was divided between secondary school and higher education levels. Nationalities were diverse, including three Americans, two Europeans, and an Asian female gamer. They usually played a daily average of 7 h, using preferably (ordered from higher to lower): only PC online games (*n* = 4), and the remaining using other platforms. Their preferred games (presented in descending order) were: MMORPGs, RPGs, FPSs, MOBAs, casual games, action adventures, simulations, and fighting games. Specifically, they played the following videogames*: The Binding of Isaac Rebirth*, *Bioshock*, *Overwatch*, *The Witcher 3*, and *World of Warcraft*.

In general, these female gamers considered the content of their favorite games to be above average regarding violence, but they had different opinions regarding whether these games were violent against women or men. Regarding their gaming patterns, four played with others online very often and the other two quite often. Interestingly, all potentially addicted participants were introduced to playing videogames by their family members (i.e., four by their fathers). Lastly, item seven was the most frequently endorsed (*n* = 6), which refers to the addiction symptoms of deceit or covering-up, while item nine was the least endorsed (*n* = 3), referring to conflict caused by the gaming behavior; all others were highly endorsed (items 3, 4, 5 and 8: *n* = 5; items 1, 2, and 6: *n* = 4).

## Discussion

The objectives of the present study were to profile female gamers internationally, determine potential predictors associated with perceived online gaming addiction, and to identify those who potentially are at risk of developing IGD. The present study fills the gap in knowledge on female gaming through utilizing a quantitative methodological approach, supporting the idea that females play popular videogames, and a small proportion of female gamers experience symptoms of IGD (American Psychiatric Association [APA], 2013) and (online) gaming disorder (World Health Organization [WHO], 2018). Furthermore, in female gamers in the present study, IGD was associated with motivations to play games (i.e., achievement and social motivations), as well as embodied presence and identification with the avatar when playing popular videogames (i.e., MOBAs, MMORPGs, and FPSs). However, masking gender using their gaming avatar did not predict IGD in the present study. In addition to this, psychopathological symptoms predicted IGD, namely hostility and social phobia, as well as a negative body image.

Considering the profile of an addicted female gamer, the analysis showed they were young adults who play popular online videogames (e.g., MMORPGs, RPGs, FPSs, and MOBAs). These games are considered to include above average violent content and exaggerated characters (i.e., hyper-sexualized female characters). Furthermore, female gamers experienced almost all addiction symptoms, especially deception, followed by (i) tolerance, loss of control, loss of interests, and escape, and (ii) preoccupation, withdrawal, continued use despite problems, whilst experiencing conflict was common only in half of them. This latter aspect, also observed in a previous study (Lopez-Fernandez, 2018), indicates empirical studies on IGD do not accurately measure addiction. According to Charlton and Danforth (2007), the reason may be that core criteria for online game playing are conflict, withdrawal, relapse, and behavioral salience, and they are not the most commonly observed criteria in potentially addicted online female gamers, although the correspondence is quite high (between 50 and 66% of them experienced these symptoms as their main symptoms).

### Establishing International Female Gamer Profiles

Regarding the first aim of the present research, female gamers are a population that requires additional research in scientific and clinical studies, because they increasingly play videogames (Entertainment Software Association [ESA], 2018; Interactive Software Federation of Europe [ISFE], 2018) and they are under-studied as a single population group. The findings of the present study are based on populations in Western continents, but a psychometric tool has been used (i.e., IGDS9-SF) which has been validated across many countries (Pontes et al., 2017; Palo et al., 2018), including validations in some of the countries represented in the present study. Female gamers play all types of game genres (especially popular online games) using different devices (i.e., PCs and consoles). Previous research suggested that female gamers play across gaming genres and use different platforms (Lewis and Griffiths, 2011), a finding that has been replicated in the present study.

Regarding sociodemographic variables as predictors of online gaming addiction, low education levels (i.e., primary and secondary levels achieved) and increased leisure time (or freedom to invest time into gaming) due to occupational situation (i.e., being unemployed or a student) was found to be associated with higher mean scores on the IGD measures. This finding seems congruent with European and Asian results on problematic online gaming including both genders (Seok and DaCosta, 2012; Lopez-Fernandez, 2018). However, the relationship between IGD and education (Lopez-Fernandez et al., 2014) as well as occupation has been scantly researched in females, and when studied, no predictors were found explaining this risky behavior (Ko et al., 2005). In summary, this research indicates that female gamers play popular online games around the world.

### Female Gamer Predictors for Internet Gaming Disorder

Concerning the second aim, several predictors emerged for potential online gaming addiction for female gamers. The most unexpected findings were achievement and social motivations were detected as sole predictors, and not immersion. Previously immersion has been found to be the online gaming motivation that predicts online gaming addiction in gamers (McLean and Griffiths, 2013; Billieux et al., 2015), as it amplifies the effects of content of virtual contexts such as those in videogames (Weinstein et al., 2009). However, in the present study, immersion was not a motivation to play videogames excessively (or potentially addictively), which is contrary to previous research on female gamers (McLean and Griffiths, 2013). The reasons for this may be that the present population was sampled using a quantitative approach with an international sample, and videogame behaviors in females have changed worldwide since the publication of the previously cited qualitative study. Women are not only playing more videogames, including popular online videogames, but they have become better gamers who compete against others (Laconi et al., 2017; Lopez-Fernandez, 2018).

Moreover, the present findings seem to show a type of immersion appears as a predictor of IGD regarding the avatar (i.e., embodied presence and identification with the gaming character) for playing popular MOBAs, MMORPGs, and FPS games. However, the predictors related to avatar identification and embodiment could explain female body image perception as the creation of an avatar requires the construction of a virtual gender, which could take sexuality into account [i.e., body image dissatisfaction (BID)]. These predictors initially did not seem to be related with a coping strategy for gender issues (i.e., coping was not a predictor in this study), make sense when considering masking other body, gender, and sexual issues which the female gamer considers to be negative (e.g., BID). For instance, female gamers want to be beautiful and powerful, so they combine different characteristics into their creation of themselves inside the game (Eklund, 2011), which Royse et al. (2007) named “embodied femininity.” At the same time, female gamers may act in a masculine way [i.e., choosing characteristics which are usually less accessible to women offline, as they want to be women on their terms within the game (Eklund, 2011)].

The association between negative body image of female gamers and online gaming addiction can be explained by the connection between their perception of violence being higher in popular online games which they usually play, and the exaggerated characters they play in these games. Research has shown the relationship between idealized and hyper-idealized bodies and individuals’ BID (Matthews et al., 2016) can be explained by two main reasons: (i) social comparison can result in upward or downward comparisons, as different virtual bodies may cause divergent body image-related outcomes, and (ii) videogame content is influenced by sexist tastes (i.e., male and female portrayals in games tend to align with heteronormative male fantasies depicting strong, capable men and highly sexualized women). This phenomenon could affect both genders differently as content appeals predominantly to a type of man [e.g., males from Western cultures who are primarily young, heterosexual, White/Anglo men (Shaw, 2011)]. Similarly, videogame content being influenced by sexist tastes may affect females, who are not so young [e.g., early adults, heterosexual, or White/Anglo women (Eklund, 2011)]. This finding is crucial as it contributes to the scarce literature on the effects of game genre characters and contents on BID in female gamers, which could be tackled in future clinical research and prospective preventive health actions.

Furthermore, two comorbid disorders appeared as predictors of IGD: hostility (Yen et al., 2011; Stavropoulos et al., 2017), and social phobia (Wei et al., 2012; Sioni et al., 2017), which have also been observed in addicted male gamers who play MMORPGs. However, depression did not emerge as a predictor, although it usually is a predictor of IGD in gamers (Laconi et al., 2017), and has been found in male gaming addicts (Wei et al., 2012) and female gamers (Wang et al., 2018). Depression frequently affects women, but not in the present study, contrary to previous research (Laconi et al., 2015). Therefore, it could be possible that gaming is used as a coping mechanism in women to deal with potential feelings of depression (although they may be unaware of these feelings), to the extent that those initial feelings are alleviated considerably, making depression a non-significant predictor of addictive gaming. This conjecture requires testing in future research. Lastly, although comorbidity seems a norm in internet addiction and clinical IGD research (Kuss and Lopez-Fernandez, 2016), in the present study hostility and social phobia are only two disorders which could explain why those females who tend to show online gaming disorder also tend to show higher irritability and avoid sharing thoughts and time with others.

### Identifying Female Gamers Who May Be at Risk for Developing Online Gaming Addiction

With respect to the third aim, gamers who play popular online games (including MMORPGs and MOBAs) are at higher risk to develop gaming addiction, as these kinds of games require time and energy invested into guilds, competition, and continuous connection, for the sake of improved performance (Kuss, 2013a; Lopez-Fernandez, 2018). The same applies to female gamers (Ko et al., 2005), as the present study found that female gamers who were considered potentially addicted to online gaming showed similar videogame usage patterns, including multiplatform gaming. This finding is contrary to previous qualitative studies on female gamers, who were considered casual gamers (Lewis and Griffiths, 2011; McLean and Griffiths, 2013), although it is worth noting that this research showed females play across different game genres using various devices (Lewis and Griffiths, 2011; Lopez-Fernandez, 2018). The only concern of these findings is the main prevalent symptoms in potentially addicted female gamers are those usually used to categorize high-engagement rather than addiction (e.g., conflict) according to Charlton and Danforth (2007), and theses have been corroborated by other subsequent studies (Seok and DaCosta, 2012; Lehenbauer-Baum and Fohringer, 2015;Lopez-Fernandez, 2018).

In the present study, female gamers based in Europe obtained lower levels of IGD, which is partially congruent with the few exiting cross-cultural studies on potential Internet and mobile phone addiction (Durkee et al., 2012; Tsitsika et al., 2014; Lopez-Fernandez et al., 2017; Laconi et al., 2018). It indicates that males are more prone to present a higher prevalence of problematic Internet use when playing videogames as adolescents, while females usually have higher Internet addiction rates due to their increased engagement in social networking (Lopez-Fernandez, 2015; Kuss and Griffiths, 2017). However, with increased age, women are more prone to higher levels of potential gaming addiction similar to men (Lopez-Fernandez, 2018). This reversed phenomenon (i.e., both genders engaging in gaming and being similarly at risk for potential gaming addiction) has been found in studies on internet use-related addiction and may be due to the change in gaming behavior during the last decade (2010–2018), where more women play videogames, and female gamers’ profiles have changed (i.e., they are now considered to be power or moderate gamers, and non-gamers; Royse et al., 2017).

Furthermore, the only cross-cultural European study conducted on gaming disorder in adolescents (Müller et al., 2015) corroborated the prevalence of IGD was very low (1.6%, instead of one per cent in the current study), higher in boys who play MMORPGs, FPSs and strategy games by themselves, which is partially congruent with the present findings related to games played. However, the extracted findings regarding IGD prevalence in adolescents seem to be slightly higher than those in adulthood, which is contrary to what has been observed in one of the few life-span studies (Festl et al., 2013), where the proportion of gaming addiction (0.2%) was stable in adolescents, younger and older adults, suggesting different age groups may be impacted by IGD similarly.

### Violence Perceived by Female Gamers

Regarding the level of violence perceived by female gamers with self-reported gaming addiction, previous studies found violent videogame play was positively related to aggressive behavior and delinquency offline; men had a more hostile view of the world than women did (Anderson and Dill, 2000). Furthermore, female characters appear to be absent in online games, or when they are present, they are highly sexualized. Violence was observed in half of the videogames, and in a quarter of these games violence was directed toward women (Dietz, 1998). However, these findings should be revised as current literature on gaming and aggression has changed considerably (e.g., more characters are females, but they are still hypersexualized or objectified), and aggression against them is still reported and is potentially causing harm.

Regarding aggression, Beck and Rose (2018) state: “instead of asking if videogames influence attitudes and behavior, perhaps we should be focusing on why videogames depicting virtual sexual objectification and violent victimization of females are culturally popular” (p. 22). In the present study, however, female gamers with a higher risk of gaming addiction were not prone to swap their gender when playing their avatar to avoid violence or aggression or to cope with stressful situations, the way adolescents seem to do (Griffiths et al., 2004). Thus, although videogames which promote this type of sexual objectification are more popular, it seems females who heavily play online games do not respond to the violence contained in these videogames (i.e., by modifying their gender in the game). However, female gamers perceive violence to be part of the games they play.

### Limitations

Despite the present study’s strength in its originality and in producing novel findings in female gamer research, it is not without limitations.

First, and most importantly, it relied on a relatively large convenience sample of female gamers across all continents and countries studied. Due to this diversity this might have affected the overall generalizability of the present findings. Furthermore, the present study included data from online forums, and is thus representative of virtual gaming culture. Recruitment was performed solely online, and therefore the study relied on participants’ self-reported responses in relation to their gender and all questions and questionnaires included. To the best of the authors’ knowledge, no prior study has ever carried out a cross-sectional exploration about this phenomenon and this strategy provided us with the opportunity of collecting data worldwide from female gamers. Therefore, the present study increases our understanding of gender issues related to women who play videogames in the present day and age.

Furthermore, all the measures were based on questionnaires administered via an online survey, which could have produced biases (e.g., social desirability or recall biases), which may affect external validity. However, this quantitative cross-sectional research on female gamer behaviors and their risk of developing IGD is helpful given the lack of other similar studies and the recent recognition of gaming disorder by the World Health Organization [WHO] (2018). The only restriction to identify potential female gamer addicts has been the conservative cut-off point used, but the original authors’ recommendations have been maintained. Nevertheless, it is suggested to use a less conservative cut-off point in future research (i.e., considering scores 4 “Agree” and 5 “Strongly agree” together, and not only 5; as other studies have done; e.g., Lopez-Fernandez, 2018), especially now that gaming disorder has been officially recognized (World Health Organization [WHO], 2018). More research is needed to be able to diagnose and treat addicted female gamers in clinical settings.

## Conclusion

This study has indicated females are a growing gamer population who enjoy playing popular online games. For a very small minority of them, online gaming addiction may be a problem, especially for those who play MMORPGs, FPSs, and MOBAs for social and achievement-related motivations, who appear to be more hostile and socially phobic, and who have a more negative body image. Female gamers are still understudied in this new virtual landscape, and future research should consider this population in more depth and breadth, especially regarding the characteristics associated with problematic play, given they are a growing population, who, similar to their male counterparts, may experience symptoms of psychopathology related to their gaming behaviors.

## Ethics Statement

This study was approved by the School of Social Sciences’ ethical review board at Nottingham Trent University (United Kingdom). Informed consent was obtained from all the participants within the online survey, on the first online page (i.e., sociodemographic questions), through confirming seven questions (e.g., “I understand the study and agree to take part in it,” “I understand my participation is voluntary, and that I am free to withdraw (…),” “I confirm that I am a woman,” “I confirm that I am 18(…),” “I confirm that I play video games,” “I confirm that I know full participant anonymity will be assured (…),” and “I know that anonymised results may also be disseminated in academic work”).

## Author Contributions

OL-F contributed to the conceptualization, formal analysis, funding acquisition, project administration, resources, and supervision. OL-F and AW performed the data curation and methodology. OL-F, DK, and AW investigated the study. OL-F drafted the original manuscript for section “Introduction,” “Materials and Methods,” “Results,” and “Discussion” and AW drafted the section “Materials and Method”. OL-F and DK reviewed and edited the manuscript.

## Conflict of Interest Statement

The authors declare that the research was conducted in the absence of any commercial or financial relationships that could be construed as a potential conflict of interest.
